# Neurexan Prescription Is Associated with Lower Risk of Sleep Disorder Recurrence and Depression Prevalence as Compared to Z-Drugs and Benzodiazepines: A Retrospective Database Analysis in Germany

**DOI:** 10.3390/healthcare12141413

**Published:** 2024-07-16

**Authors:** Göran Hajak, Céline Vetter, Martin Wehling

**Affiliations:** 1 Clinic for Psychiatry, Psychosomatic Medicine and Psychotherapy, Sozialstiftung Social Foundation Bamberg, Teaching Hospital of the University of Erlangen, 96049 Bamberg, Germany; 2 IQVIA Commercial GmbH & Co. KG, 60549 Frankfurt am Main, Germany; 3 Medical Faculty Mannheim, University of Heidelberg, 68167 Mannheim, Germany

**Keywords:** real-world evidence, natural medicinal product, hypnotics, sleep disorder, nervous restlessness, stress, database analysis, retrospective cohort study

## Abstract

Real-world evidence on the association between natural medicinal products and the recurrence of sleep disorders is currently limited, particularly when compared to the evidence reported for prescription hypnotics. In a retrospective cohort analysis, we investigated patients with sleep disorders prescribed either the natural medicinal product Neurexan (Nx4), benzodiazepines, or nonbenzodiazepines (Z-drugs) using the IQVIA Disease Analyzer database, which encompasses electronic medical records nationwide in Germany. A 1:1 matching procedure based on age, sex, prevalent depression, anxiety or adjustment disorder, and the number of medical consultations in the past 12 months resulted in four cohorts: patients prescribed Nx4 were matched with those prescribed Z-drugs (two cohorts with 8594 matched patients each), and another cohort of patients prescribed Nx4 were matched with those prescribed benzodiazepines (7779 matched pairs). Results from multivariable-adjusted Cox regression models demonstrated that Nx4 was associated with a significantly lower risk of recurrent sleep disorder diagnosis within 30–365 days after prescription compared to both Z-drugs (HR = 0.65, 95%CI = 0.60–0.70, *p* < 0.001) and benzodiazepines (HR = 0.85, 95%CI = 0.79–0.93, *p* < 0.001). Additionally, Nx4 was associated with a lower prevalence of depression compared to Z-drugs (HR = 0.90, 95%CI = 0.83–0.98, *p* = 0.020) and benzodiazepines (HR = 0.89, 95%CI = 0.82–0.97, *p* = 0.009). These findings suggest an association between Nx4 and improved sleep and mental health outcomes. However, due to inherent limitations in the study design, the causality of this relationship cannot be stated.

## 1. Introduction

Global psychosocial stressors such as the SARS-CoV-2 pandemic, the Russia–Ukraine war, and the decline of the economy have significantly affected individual well-being, with problems sleeping being the mental seismograph for an altered coping ability. Worldwide, 1 out of 4 adults suffers from significant difficulties initiating or maintaining sleep with a negative impact on daytime functioning and well-being [1]. Approximately 40% of healthcare workers experience insomnia [2]. At least 1 in 3 adults in Germany reports symptoms consistent with sleep disorders, such as insomnia, the most common sleep disorder [3]. Data collected amongst German adult employees in 2017 indicate that up to 80% of adults report sleep problems, and approximately 10% of employees report severe sleep disorders such as insomnia [4]. The implications for public health are becoming increasingly evident, as the importance of sleep for health and well-being is highlighted by a growing body of literature. Specifically, insomnia is the number one contributor to work absenteeism [5] and workplace errors [6] and is highly associated with workplace and non-workplace injuries [7]. Insomnia is also associated with an increased risk of daytime fatigue and accidents, mood disorders, and chronic diseases such as certain cancers, cardiovascular disease, metabolic disorders, and neurodegenerative disorders [8,9,10,11,12,13]. Insomnia is one of the most common sleep disorders and is linked to a complex, multi-factorial pathophysiology, interfering with the complex neuro-orchestrating of sleep–wake regulation [14]. Several lines of evidence suggest that patients with insomnia suffer from central nervous system hyperactivity during the transition from wake to sleep and during sleep, exhibiting psychophysiological hyperarousal as the disease-determining key feature [15]. Nervous restlessness, worries, uncontrollable circles of thoughts, and their concomitant emotional symptoms thereby contribute critically to disease etiology [16]. Key symptoms include increased sleep latency and wakefulness after sleep onset. Various pathways are used to treat insomnia in clinical practice. Among sleep experts, cognitive behavioral therapy (CBT) for insomnia is ranked as the number one treatment and recommended in guidelines worldwide, and it may be used in combination with hypnotic drugs [17,18]. However, the limited availability of CBT prevents extensive application [19]. Meta-analyses have shown a wide range of prescription-only medications to reliably promote sleep in the short-term [20,21,22], but their unfavorable benefit–risk ratio and lack or neglect of long-term treatment data limit their use [23,24]. A survey of the pharmacological treatment landscape in chronic insomnia in five European countries (France, Germany, Italy, Spain, and the United Kingdom) suggests that benzodiazepines (BZD) and nonbenzodiazepines (Z-drugs such as zaleplon, zolpidem, zopiclone, eszopiclone) are the most widely used treatments in chronic insomnia and are being used for longer than their recommended duration [25]. These drugs act as GABAA receptor-positive allosteric modulators of the BZD site and promote sleep by potentiating gamma-aminobutyric acid (GABA) activity [26], the principal inhibitory neurotransmitter in the human brain [27]. Prescription data also show that the long-term use of BZD is associated with multiple consequences of treatment, including dependence, but also that previous use of BZD may increase the risk of opioid use disorder [25] and that long-term use of BZD may be correlated with Alzheimer’s disease [28]. Withdrawal from those medications can lead to serious and potentially life-threatening withdrawal symptoms if intake is abruptly discontinued [29]. Therefore, most guidelines recommend limiting the use of these drugs to three to four weeks maximum [24]. From 2006 to 2015, 1.64 million patients in Germany received prescriptions for BZD or Z-drugs according to statutory health insurance claims data, with about one-third of patients receiving prescriptions that could be qualified as “risky”, i.e., long-term or high-dose prescriptions [30]. Especially in the elderly, BZD and Z-drugs can cause clinically relevant adverse cognitive and psychomotor events as well as daytime fatigue [31]. In a recent consensus statement, the European Geriatric Medicine Society unanimously identified BZD and Z-drugs as fall risk-increasing drugs [32]. These and other findings have led to BZD and Z-drugs being labeled as potentially risky medications that should be avoided in older patients [33,34,35,36,37]. Over-the-counter (OTC) natural medicinal products such as Neurexan^®^ (Nx4), which has been demonstrated to reduce the stress response and thereby may have beneficial effects on sleep, represent an alternative to prescription-only hypnotics for patients with insomnia symptoms. Though no prescription is needed to obtain Nx4 in a pharmacy, doctors may still prescribe such OTC medicinal products as a treatment recommendation and/or on patient request. Nx4 is a homeopathically manufactured non-prescription medicinal product approved by the German Federal Institute for Drugs and Medical Devices (Bundesinstitut für Arzneimittel und Medizinprodukte [BfArM]) for addressing nervous restlessness and associated sleep disorders. Its active ingredients include *Passiflora incarnata* (passionflower), *Avena sativa* (oats), *Coffea arabica* (coffee), and *Zincum isovalerianicum* (zinc valerianate); it is available in tablet and drop form. A placebo-controlled, double-blinded, randomized trial showed that Nx4 significantly reduced salivary cortisol as well as plasma adrenaline after acute psychological stress exposure as compared to placebo [38]. This trial also found that the safety profile of Nx4 was comparable to that of placebo. Further placebo-controlled trials have demonstrated that Nx4 is associated with attenuation of the stress response and reduced stress-induced hyperactivation in brain areas processing emotions, vigilance, attention, and the brain at rest as assessed by neuroimaging and electrophysiology [39,40,41,42,43]. Finally, a non-interventional study examined the association of Nx4 and valerian—an herbal medicinal product considered likely to improve sleep [44]—with sleep duration and latency in 409 patients with mild to moderate insomnia. The authors observed that both Nx4 and valerian increased sleep duration and reduced sleep latency to a comparable extent [45]. Despite the evidence that natural medicinal products such as Nx4 may reduce nervous restlessness and thus might also be useful in the treatment of patients with sleep disorders, real-world data are currently scarce. Thus, the aim of the present study was to examine the association between the prescription of Nx4 and the recurrence of sleep disorder diagnoses or prevalence of depression [46,47] compared to Z-drugs and BZD in patients within the IQVIA Disease Analyzer (DA) database. This electronic medical record (EMR) database has been shown to be representative of the population in Germany [48,49].

## 2. Materials and Methods

### 2.1. Data Source

The analyses utilized the IQVIA DA database, encompassing case-based information from office-based physicians, comprising both general practitioners (GPs) and specialists in Germany. This database incorporates details on patient demographics, drug prescriptions, concomitant medications, comorbidities, sick leave, and referrals. Specifically, the database carries information provided by 3000 office-based physicians, representing 3–5% of all practices in Germany. Practices can be categorized into ten classes based on the physician’s specialty (GPs and various specialists). The sample of practices included in the DA database is representative of Germany as a whole and is suitable for pharmacoepidemiological and pharmacoeconomic studies [48,49]. Data in the DA database are anonymized in compliance with the applicable legislation. For the present type of retrospective study involving anonymized data, no ethics committee approval was required. Neither the authors involved, IQVIA, nor the company funding the research had access to any identifying information at any time. IQVIA ensures the accuracy, consistency, and completeness of the data through regular monitoring.

### 2.2. Study Population

**Figure 1:**
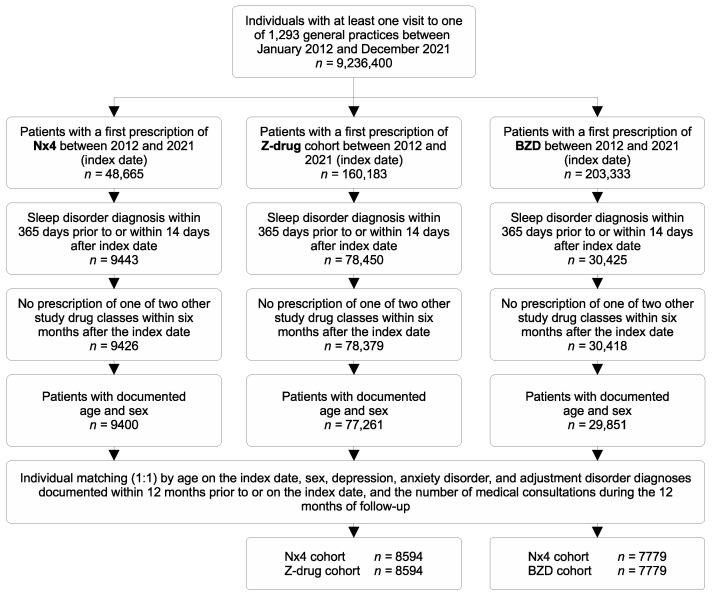
Cohort selection. Four study cohorts were selected for this study to compare sleep disorder recurrence and depression diagnosis prevalence in patients with sleep disorders (with any ICD-10 diagnoses of G47 or F51) treated with Neurexan (Nx4) as compared to Z-drugs (final cohorts of matched pairs: *n* = 8594) and benzodiazepines (BZD; final cohorts of matched pairs: *n* = 7779). Patients were selected from the IQVIA Disease Analyzer (DA) database, which comprised over 9.2 million patients between January 2012 and December 2021 and is representative of the German population [49].

This study included patients in general practices in Germany who received a prescription for Nx4, Z-drugs, or BZD between January 2012 and December 2021. The index date was defined as the first date on which the respective prescriptions were issued. In general, patients were only included if they had been diagnosed with a sleep disorder according to the classification of the World Health Organization [50] (i.e., ICD-10 codes G47, disorders of initiating and maintaining sleep [insomnia], or F51, non-organic sleep disorder), in the 365 days prior to, or the 14 days after, the index date. Patients were excluded if they had been prescribed more than one study treatment (i.e., Nx4, Z-drugs, or BZD) at the same time on the index date or within six months after the index date. If patients were prescribed multiple study drugs more than six months apart, the earliest prescription was used as the index prescription. Each patient could only be selected once and contribute to either the Nx4, Z-drug, or BZD cohorts. Other sleep aids or therapies outside of the study treatments were not considered for inclusion or exclusion criteria. Specifically, out of all individuals with at least one visit to one of the 1293 general practices in the database between January 2012 and December 2021 (*n* = 9,236,400; Figure 1), 48,665 patients with a first Nx4 prescription between 2012 and 2021 were selected. Out of those, 9443 had a sleep disorder diagnosis (G47 or F51). Most patients receiving Nx4 had no other study drug within 6 months after their index date and were selected going forward (*n* = 9426). Only 26 patients were missing age and sex information, such that 9400 Nx4 patients were selected for matching procedures. The same criteria were applied to the Z-drug and BZD cohorts. Overall, age and sex were missing in <2% of patients within the selected cohorts of the DA database. Patients receiving Nx4 were then matched (1:1, nearest neighbor matching) to those with a Z-drug prescription by age on the index date; sex, depression (F32 or F33), anxiety disorder (F41), or reaction to severe stress/adjustment disorder (F45) diagnoses documented within 12 months prior to or on the index date; and the number of medical consultations during the 12 months of follow-up. In total, 8594 patients prescribed Nx4 were successfully matched with 8594 patients prescribed Z-drugs from a pool of 77,261 eligible Z-drug patients. The same matched–pair process was repeated to match Nx4 patients with patients with BZD prescriptions (Figure 1), and 7779 patient pairs were matched out of 9400 Nx4 patients and 29,851 BZD patients. Where several BZD or Z-drug patients were eligible for matching, the matched patient was randomly chosen. Note that this matching procedure resulted in an overlap between the two cohorts of Nx4 patients being matched with Z-drug and BZD patients.

### 2.3. Endpoints and Statistical Methods

To evaluate matching success, descriptive statistics were used to compare cohort characteristics between the Nx4 versus Z-drug cohorts and the Nx4 versus BZD cohorts using the Wilcoxon signed-rank test for continuous variables, the McNemar test for categorical variables with two categories, and the Stuart–Maxwell test for categorical variables with more than two categories. The association between study treatments and recurrence of sleep disorder diagnosis (i.e., recurrence of ICD-10 codes G47 or F51 within 30–365 days after the index date) was then tested using Cox regression models. This means that in addition to the required sleep disorder, diagnosis within 365 days prior to or within 14 days after the index date (i.e., Nx4, Z-drug, or BDZ prescription date), a re-coding of the sleep disorder diagnosis within 30–365 days post-index was necessary for a patient to be counted as having a recurrent sleep disorder. Recurrence of sleep disorder diagnosis was chosen as the primary endpoint, as its reduction is one of the most important outcomes of insomnia treatment and may be a key factor for long-term pharmacotherapy [51,52]. Hazard ratios (HRs) with 95% confidence interval (95%CI) were estimated with the Z-drug and BZD cohorts as reference groups. Models were adjusted for predefined common co-diagnoses (including diabetes mellitus, back pain, chronic heart diseases, chronic bronchitis, chronic obstructive lung disease (COPD), and cancer) that might confound the association between the study treatment and sleep disorder recurrence. No additional adjustments were made, as patients between cohorts were matched 1:1. *p*-values < 0.05 were considered statistically significant. Secondary analyses examined the association between the study treatment and depression prevalence (ICD-10 code: F32 = Depressive episode). As sleep disorders can be part of the presentation of a mood disorder, but can also precede the development of mood disorders [47,53,54,55], all existing cases of depression were included—both those present at the time of study inclusion and those occurring throughout the follow-up period. As patients were matched by depression status on the index date, there were no group differences in this respect between cohorts at the time of study inclusion. Thus, all differences in depression prevalence post-index date would be due to a new depression diagnosis or the recurrence of a depression diagnosis that had initially occurred prior to the 12-month look-back window. The proportion of patients with recurrent sleep disorders, as well as depression diagnoses between 30 and 365 days after the index date, was also evaluated using Kaplan–Meier curves to gain insights into the potential temporal differences in sleep disorder recurrence between groups. In addition, secondary analyses also addressed the question of physicians’ preference to prescribe one of the study drugs. Disease severity information is not available as part of the database, but since BZD and Z-drugs are generally prescribed for more severe cases, it is a likely confounder in these analyses. One would expect similar proportions of the study drugs by practice if disease severity were the main predictor of study drug selection. The proportion of patients to whom physicians prescribed Nx4, Z-drugs, or BZD was also examined. Imbalances in the proportions of study drug prescriptions would indicate that study treatment was a function of physician preference, rather than (only) disease severity. Exploratory stratified analyses examined whether the association between study treatments, sleep disorder recurrence, and depression diagnosis differed (i) between men and women and (ii) by age group. In secondary post-hoc analyses, we included additional variables in the matching process to assess the overall robustness of our results. Specifically, we aimed to reduce selection bias and the impact of potential confounders on the examined associations. Initially, nearest neighbor propensity scores were based on age at the index date; sex; diagnoses of depression (F.32 or F.33), anxiety disorder (F.41), or reaction to severe stress/adjustment disorder (F.45) documented within 12 months before or on the index date; and the number of medical consultations during the 12 months of follow-up. A standardized mean difference (SMD) of less than 0.1 was allowed indicating that adequate covariate balance has been achieved. The matching process was conducted separately to match patients receiving Nx4 with those prescribed Z-drugs or BZDs. Additional variables were sequentially included in the matching algorithm: co-morbidity diagnoses (including diabetes [E.10, E.11, or E.14], COPD [J.42, J.43, or J.44], back pain [M.54], cancer [any diagnostic C code], and heart disease [I.2, I.3, I.4, or I.5]), antidepressant prescriptions within 12 months before or on the index date, and practice prescription behavior. Practice prescription behavior was estimated based on the number of patients with sleep disorders who received Nx4 versus Z-drugs or benzodiazepines. The proportion of patients per practice was calculated based on the number of patients who received Nx4 (0%, 1–24%, 25–49%, 50–79%, and ≥80%). Further exploratory analyses examined whether the study treatments differed in terms of their safety profiles within days 1–365 after the index date, i.e., whether patients with Nx4 prescriptions had a lower prevalence of fractures (ICD-10 codes S02, S12, S22, S42, S52, S62, S72, S82, S92, T02, T08, T10, T12) or lower levels of respiratory tract infections (J00–J06), gastrointestinal symptoms (ICD-10 codes: K59, R10–R15), or cognitive symptoms (including somnolence, dizziness, syncope, or memory disorders, ICD-10 codes: R40, R41, R42) than patients on Z-drugs or BZD. It is worth noting that GP practices in Germany tend to underreport potential side effects, and they might document diagnoses without including a link to a given therapy. Thus, examining safety profiles using EMR, as was the case in this study, is limited but might complete the picture with regard to the link between study treatments and patient outcomes. In general, the goal was to determine whether associations with Nx4 would extend to the absence of known side effects of both Z-drugs and BZD. All analyses were conducted with SAS Version 9.4.

## 3. Results

### 3.1. Baseline Characteristics

A total of 8594 Nx4 patients were matched with an equal number of Z-drug patients and a total of 7779 Nx4 patients were matched with an equal number of BZD patients. Baseline characteristics of the study cohorts are shown in Table 1. In the Nx4 versus Z-drug analysis, patients were 48.0 (SD = 18.5) years old on average, and 62.5% were female. A total of 14.9% of the matched Nx4/Z-drug patients had been diagnosed with depression prior to the index date, 5.1% with anxiety disorder (ICD-10 F41), and 15.8% with reaction to severe stress and adjustment disorders (ICD-10 F43). The prevalence of back pain (ICD-10 M54) was slightly higher in the Nx4 than in the Z-drug cohort, while diabetes (ICD-10 E10, E11, E14) and cancer (ICD-10 C codes) were more often diagnosed in the Z-drug than in the Nx4 cohort. In the comparison of outcomes between Nx4 and BZD patients, the average age of patients was 50.0 years (SD = 17.9), with 63.0% being female. Among these patients, 15.9% were diagnosed with depression, 6.6% with an anxiety disorder, and 14.1% with reactions to severe stress and adjustment disorders. The Nx4 cohort exhibited a slightly higher prevalence of back pain, while diabetes, COPD (ICD-10 J42-44), and cancer were more frequently diagnosed in the BZD cohort (Table 1).

### 3.2. Nx4 Is Associated with a Significantly Lower Recurrence of Sleep Disorder Diagnosis as Compared to Z-Drugs and BZD

**Figure 2:**
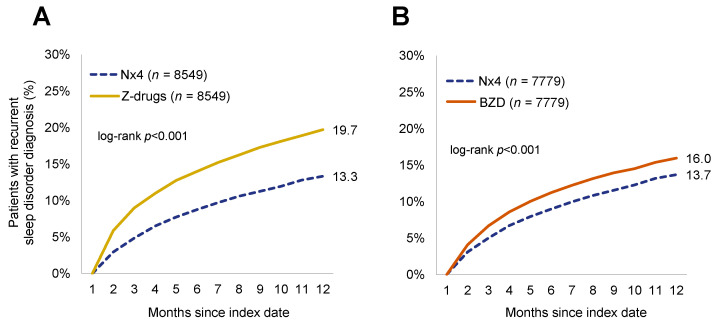
Cumulative incidence of recurrent sleep disorder diagnosis. (**A**) Patients with Neurexan (Nx4) versus Z-Drug prescriptions. After 12 months, cumulative incidences were at 19.7% for Z-drug versus 13.3% for Nx4 (*p* < 0.001; Kaplan–Meier log-rank test, unadjusted). (**B**) Patients with Nx4 versus benzodiazepine (BZD) prescriptions. After 12 months, cumulative incidences were at 16% for BZD versus 13.7% for Nx4 (*p* < 0.001; Kaplan–Meier log-rank test, unadjusted).

Within 30 days and 12 months after the index date, a total of 13.3% of Nx4 patients had a documented recurrent sleep disorder diagnosis as compared to 19.7% of Z-drug patients and 16.0% of BZD patients (*p* < 0.001 for both comparisons) (Figure 2).

Cox regression analyses showed that the Nx4 cohort had a 35% lower recurrence of sleep disorder diagnosis than the Z-drug cohort (HR = 0.65; 95%CI = 0.60–0.70, *p* < 0.001). When comparing the Nx4 and BZD cohorts, Nx4 prescription was associated with a 15% lower recurrence of sleep disorder diagnosis than patients with BZD prescriptions (HR = 0.85; 95%CI: 0.79–0.93, *p* < 0.001, see Table 2).

**Figure 3:**
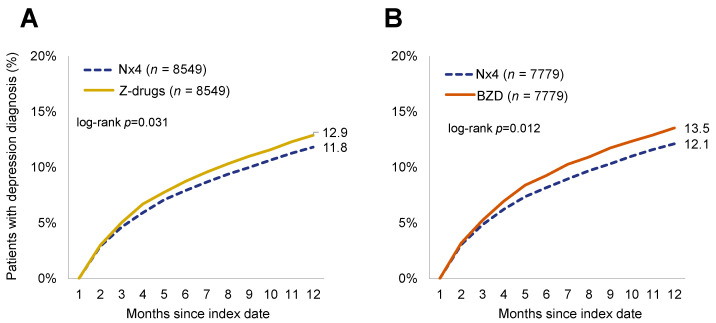
Cumulative prevalence of depression diagnosis. (**A**) Patients with Neurexan (Nx4) versus Z-drug prescriptions. After 12 months, cumulative prevalences were at 12.9% for Z-drug versus 11.8% for Nx4 (*p* = 0.031; Kaplan–Meier log-rank test, unadjusted). (**B**) Patients with Neurexan (Nx4) versus benzodiazepine (BZD) prescriptions. After 12 months, cumulative prevalences were at 13.5% for BZD versus 12.1% for Nx4 (*p* = 0.012; Kaplan–Meier log-rank test, unadjusted).

Stratified analyses showed that associations were similar in men and women and remained statistically significant despite smaller sample sizes within each stratum. Similarly, the age stratification showed that Nx4 was consistently associated with a lower risk of recurrent sleep disorder in each of the age categories. Effect sizes were attenuated in the strata of the 41–50 and the 61–70-year-old patients in the Nx4 versus BZD cohorts, while associations remained statistically significant in patients aged 31–40 and 51–60 years. Overall, the association with a lower risk of sleep disorder recurrence was more pronounced and more robust across subgroups when comparing Nx4 to Z-drugs than when comparing Nx4 to BZD. Nx4 patients had a significantly lower depression prevalence than Z-drug and BZD patients post-prescription. Figure 3 shows the prevalence of depression diagnoses in the Nx4, Z-drug, and BZD cohorts. In both comparisons, the proportion of patients with depression diagnosis was significantly lower in the Nx4 cohort (11.8% Nx4 versus 12.9% Z-drug, *p* = 0.031, see Figure 3A, and 12.1% Nx4 versus 13.5% BZD, *p* = 0.012, see Figure 3B) at the end of the 12-month follow-up period.

The results of the multivariable regression analyses are shown in Table 3. In the total population, Nx4 was associated with a significantly lower prevalence of depression diagnosis compared to Z-drugs (HR: 0.90; 95%CI: 0.83–0.98, *p* = 0.020) and BZD (HR: 0.89; 95%CI: 0.82–0.97, *p* = 0.009). In exploratory sex- and age-stratified subgroup analyses, associations remained similar in terms of magnitude of effect (except for the subgroup of patients aged > 70 years) but were no longer statistically significant, possibly due to the comparisons being underpowered.

### 3.3. Physician Preferences Contribute to Prescription Patterns for the Study Therapies: 63% of Practices Prescribe Nx4 to Their Patients with Sleep Disorders

**Figure 4:**
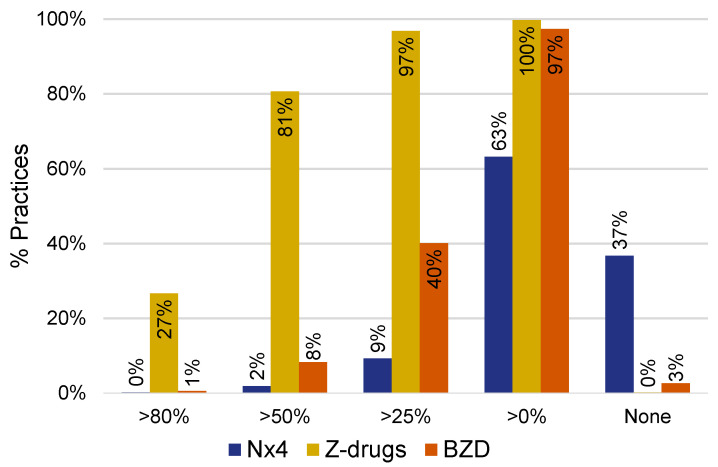
Distribution of study treatments across practices. We examined the proportion of study treatments among first prescriptions issued to patients with sleep disorders included in this study. We hypothesized that if disease severity was key to treatment selection, then the distribution of treatments across practices (*n* = 1287) should be similar. Additionally, 37% of practices did not prescribe Nx4, and 63% did.

As shown in Figure 4, out of 1287 practices, 24 (1.9%) prescribed Nx4 to more than 50% of patients with sleep disorders, while 119 (9.2%) of practices prescribed Nx4 to more than 25% of patients. Overall, 63.2% of practices prescribed Nx4 to patients presenting with sleep disorders during the study period. By comparison, only 0.2% and 2.4% of practices never prescribed Z-drugs or BZD to patients with sleep disorders during the study period. Z-drugs were the most common study treatment, with 1038 (80.7%) of practices prescribing Z-drugs to more than 50% of their patients with sleep disorders.

### 3.4. Results Remained Robust in Secondary Analyses Incorporating Potential Confounders

Additional secondary analyses tested the robustness of our results by incorporating potential confounders into the matching algorithm, such as antidepressant prescriptions (prior to or at the index date, as an indicator of depression severity) and practice prescription behavior. The results for recurrent sleep disorder diagnoses during follow-up remained robust, showing a lower probability of sleep disorder recurrence in patients prescribed Nx4 compared to patients prescribed Z-drug or BZD ($HR_{z\text{-}drugs}$ between 0.64 and 0.68, $HR_{BZD}$ between 0.81 and 0.85; all CIs < 1, *p*-values < 0.001; see Supplementary Table S5). Regarding additional adjustments for the depression analyses, the results were overall similar to those presented in Table 3. However, when adjusting for prescription preferences, the associations diminished ($HR_{z\text{-}drugs}$ = 0.98, 95%CI = 0.92–1.06, *p* = 0.664; $HR_{BZD}$ = 1.00, 95%CI = 0.92–1.11, *p* = 0.844). For a detailed overview of these secondary analysis results, please refer to Supplementary Tables S5 and S6.

### 3.5. Potentially Associated Adverse Events Show Comparable Overall Profiles across Study Therapies

Nx4 was not associated with a significantly lower prevalence of fractures as compared to Z-drugs (HR = 0.93, 95%CI = 0.76–1.12, *p* = 0.436) or BZD (HR = 0.93, 95%CI = 0.76–1.15, *p* = 0.514). No significant trends were observed across age groups for fractures, and the association remained statistically insignificant for the >70 years group ($HR_{z\text{-}drugs}$ = 0.75, 95%CI = 0.54–1.03, *p* = 0.074; $HR_{BZD}$ = 0.74, 95%CI = 0.54–1.02, *p* = 0.068; see Supplementary Table S1). There was no significant overall association between Nx4 prescription and lower respiratory tract infection prevalence ($HR_{z\text{-}drugs}$ = 0.93, 95%CI = 0.76–1.12, *p* = 0.436; $HR_{BZD}$ = 0.93, 95%CI = 0.85–1.02, *p* = 0.134, see Supplementary Table S2), although the incidence of infections in older patients (groups 51–60 and 61–70) with Nx4 prescriptions was significantly lower than in those with BZD prescriptions (51–60 years: HR = 0.79, 95%CI = 0.65–0.97, *p* = 0.022, 61–70 years: HR = 0.69, 95%CI = 0.52–0.92, *p* = 0.011; however: 71+ years: HR = 1.27, 95%CI = 1.00–1.63, *p* = 0.053). Nx4 patients were also not significantly less likely to have digestive symptoms ($HR_{z\text{-}drugs}$: HR = 1.07, 95%CI = 0.99–1.17, *p* = 0.107; $HR_{BZD}$ = 1.05, 95%CI = 0.95–1.15, *p* = 0.339, see Supplementary Table S3). Finally, we observed a significant association with a higher prevalence of symptoms involving cognition and perception among Nx4 patients compared to Z-drug patients but not BZD patients ($HR_{z\text{-}drugs}$ = 1.41, 95%CI = 1.22–1.62, *p* < 0.001; $HR_{BZD}$ = 1.05, 95%CI = 0.95–1.15, *p* = 0.339, see Supplementary Table S4).

## 4. Discussion

This real-world study examined the association between the prescription of Nx4 and the recurrence of sleep disorder diagnoses compared to Z-drugs and BZD in patients with a sleep disorder diagnosis, based on data gathered from the IQVIA DA database. A total of 63% of practices prescribed Nx4 to patients with sleep disorder diagnosis. Two cohorts of patients with a sleep disorder who were prescribed Nx4 were matched to Z-drug and BZD patients by age, sex, and comorbidities. Prescription of Nx4 was significantly associated with a lower risk of sleep disorder recurrence compared to prescription-only hypnotics, with this risk being 35% lower than in Z-drug patients and 15% lower risk than in BZD patients. An analysis of prescription habits shows that sleep disorder severity alone is unlikely to explain these results, although it is likely a confounding factor. Secondary analyses also showed that prescription of Nx4 was associated with an approximately 10% lower risk of having depression within 30–365 days post-Nx4 prescription as compared to Z-drugs and BZD. Our results, based on EMR in the outpatient setting, however, do not suggest that Nx4 is associated with fewer side effects at large compared to prescription hypnotics. There were indications, though, of beneficial effects in higher age strata, especially concerning fractures. Nevertheless, it is noteworthy that GP practices tend to underreport side effects.

### 4.1. Evidence for Lower Incidence of Sleep Disorder Recurrence in Nx4 Cohorts Compared to Z-Drug and BZD Cohorts

This study provides real-world evidence of a significantly lower incidence of sleep disorder recurrence among patients with Nx4 prescriptions compared to those prescribed Z-drugs or BZD. Because of the potentially hazardous side effects of Z-drugs and BZD, including severe withdrawal symptoms and cognitive symptoms (especially in the elderly), there is a clear need for alternative treatment options to support symptom reduction in patients with a sleep disorder. In general, clinical studies focusing on natural medicinal products are scarce. In 2010, a systematic review of randomized clinical trials of homeopathic treatments for insomnia concluded that existing randomized controlled trials were of low quality and likely underpowered [56,57]. More recent publications on natural medicinal products describe comprehensible physiological mechanisms to improve sleep [58] but still complain about insufficient study quality and heterogeneous substance groups [59], which results in an unclear picture of their effect size and benefit–risk ratio in daily use. Thus, real-world evidence represents an opportunity to understand treatment patterns and associations within representative patient populations. It is speculative but worth discussing whether Nx4’s counteracting effects on nervousness and stress-related physiology might account for the lower incidence of sleep disorder recurrence. Several lines of evidence from experimental studies support this assumption, such as the observation that Nx4 significantly reduced salivary cortisol as well as plasma adrenaline after acute psychological stress exposure in adults compared to placebo [38] and attenuated the stress response, as assessed by functional magnetic resonance neuroimaging and electrophysiology [39,40,41,42,43].

### 4.2. Lower Depression Prevalence in Patients with Nx4 Prescriptions Compared to Patients with Z-Drug and BZD Prescriptions

Our analyses also showed that depression prevalence was significantly lower in Nx4 patients 30–365 days post-prescription date compared to patients receiving prescription-only hypnotics. There are several possible explanations for this. On the one hand, it has been shown that sleep disturbances and sleep disorders such as insomnia increase the risk of depression [60], and disturbed sleep is also among the diagnostic criteria for depression [50]. While the bi-directional relationship between sleep and mood is now well established [46,48,54,55,61], little is still known about the relationship between sleep and mood in patients seeking clinical care—most studies have been conducted in healthy individuals. A recent US study examined daily mood and daily sleep quality in participants categorized (but not diagnosed) as depressed and/or anxious based on validated screening instruments and showed that the association between sleep quality and next-day mood was stronger than vice versa [62]. It is biologically plausible that improved sleep due to a reduction in nervous restlessness might also have beneficial effects on mood. On the other hand, this result may be due to lower disease severity for both outcomes, i.e., sleep disorders as well as depression, in the Nx4 patient population. While disease severity indicators are not available in the database, the prescription patterns observed in this study suggest that physician preferences rather than disease severity drive Nx4 prescription. While disease severity has to be considered as an unmeasured confounder in this study (as it is not available in German EMR data and likely a predictor of both Nx4 prescription and sleep disorders), one would assume that if severity was the key predictor of Nx4 prescription, then Nx4 prescription frequency should be equally distributed among practices. In fact, when adjusting for practice prescription preferences, we observed that the association between Nx4 and lower odds of depression was attenuated. This suggests that disease severity might indeed be an important confounder, and potentially a mediator, in the association between Nx4 use and depression prevalence following Nx4 prescription.

### 4.3. No Significant Differences in Potentially Associated Adverse Effects across Study Treatments

Our post-hoc safety analyses indicate that older patients, in particular, had a lower incidence of fractures when prescribed Nx4 compared to Z-drugs or BZD. It is well known that these prescription hypnotics can induce dizziness, fatigue, and muscle relaxation [29], and thereby increase the risk of falls and fractures in the elderly population [63,64]. Reducing falls and fractures in the elderly population through prescription practices could have significant implications for public health, such as improving the quality of life in older age, reducing hospitalizations, and lowering the cost of healthcare resource utilization. Additional real-world evidence with a focus on the safety of hypnotics and natural medicinal products is needed to further improve our understanding of pharmacological treatment options for sleep disorders and their safety implications, especially for the elderly. An alternative explanation might be that patients with Nx4 prescriptions might be at a lower risk of falling and fractures to begin with, meaning that the protective effect described might be due to reverse causation. Given the known biological and epidemiological evidence linking Z-drugs and BZD to falls and fractures, however, reverse causation is less likely to be the main explanation. A second signal identified in these exploratory analyses is that cognitive symptoms were more prevalent in patients with Nx4 prescriptions than those with Z-drug prescriptions across all age groups. In this case, reverse causation might be the most plausible explanation. Specifically, hypnotics such as Z-drugs and BZD are not recommended for cognitively impaired or vulnerable patients, since they have been identified as risk factors for incident dementia and cognitive impairment [65]. Given the overall higher prevalence of cognitive symptoms at index in the Nx4 group, especially among patients aged 51 years and older (approximately 2 percentage points higher compared to Z-drug and BZD patients), it is possible that Nx4 might have been prescribed to patients who were most likely to eventually experience cognitive and perception-related symptoms. This explanation is further corroborated by the absence of a plausible biological mechanism linking Nx4 to cognitive impairment. In addition, cognitive symptoms might be less reliable as coded EMR, especially in comparison to other evaluated symptoms, such as fractures, further limiting the reliability of this exploratory finding. Finally, it is important to acknowledge that routine care data are challenging to use for assessing safety profiles. Medications are not always linked to specific diagnoses (if at all possible for the doctor to make this causal attribution), so that underreporting of side effects is likely. Thus, the observed results should be interpreted with caution.

### 4.4. Study Limitations

This study was subject to several noteworthy limitations.

#### 4.4.1. No Insights Regarding Specific Sleep Disorders and Add-On Therapies

First, this study is limited by the fact that 80% of GPs coded sleep disorders as “unspecified sleep disorders”, meaning that it cannot offer any insights into specific sleep disorders. In addition, no information is available on add-on therapies such as OTC medications or on adherence to psychotherapy. It should be noted that while both the European Sleep Society and German clinical guidelines clearly recommend CBT for the treatment of insomnia [23,24], the EMRs do not carry information indicating whether patients are receiving this therapy or are treated in a psychotherapy setting. It is also unknown whether and to what extent patients with Nx4 prescriptions (and prescriptions for natural medicinal products in general) might be more likely to receive CBT and whether this might have introduced a potential bias. The main caveats about CBT remain the lack of adequately trained therapists and variability in terms of training available in different parts of the world [66], including Germany, which suggests a very limited impact of CBT on health in general [19].

#### 4.4.2. No Insights on the Severity of Sleep Disorders and Depression

Second, the severity of sleep disorders is not specified in the EMR used as the data source and thus could not be analyzed in the present study. It is possible that Z-drug and BZD patients had more severe symptoms, meaning that renewed documentation of sleep disorder diagnosis is more likely for these patients. However, it was shown that the prescriptions of each study drug depended instead on physicians’ preferences. Specifically, one would expect an equal distribution of sleep disorder severity among a representative sample of physician practices. Thus, the study drugs should have been equally common when analyzing prescription frequency by practice. However, a wide range of prescription patterns was observed, indicating that physician prescription preference is a contributing factor. Nevertheless, sleep disorder type and disease severity are likely confounders in these analyses that should be addressed in future studies. It is noteworthy that depression diagnosis severity was not captured in these analyses. Although the baseline prevalence of anxiety, depression, severe stress, and adjustment disorder did not differ between groups (as they were part of the matching criteria), one could argue that depression severity might have been more pronounced in patients with Z-drug or BZD prescriptions. The cohorts were matched based on the number of clinical consultations during the 12 months of follow-up. While this approach does not account for disease-specific variability between patients, it served as a useful criterion for matching general disease severity in the cohorts.

#### 4.4.3. Prescription-Based Data

Third, EMR studies, in general, are limited by the fact that they can study what is being prescribed but not what is ingested. In other words, it is not possible to control for OTC medication potentially consumed. In our case, this implies that patients with Z-drug or BZD prescriptions could also have taken Nx4 or any other non-prescription therapy. Nevertheless, this would have biased our results towards the null, and thus led to an underestimation of effect sizes rather than an overestimation.

#### 4.4.4. Limitations of Retrospective EMR Database Analyses

Data reporting may be incomplete, and relevant diagnoses or adverse drug reactions may be missing. In addition, the choice of drugs by participating practitioners may reflect personal preferences rather than medical necessity. Results cannot be considered causal but rather indicative of associations that may be used to generate hypotheses, contribute to the triangulation of findings in epidemiology [67], and inform clinical trial design and intervention studies. As the database was not constructed to specifically collect data on sleep disorders, major data on the disorders and disease-related efficacy of drugs, which are normally of interest in controlled studies, are missing. Thus, no reliable analyses of efficacy were possible using the routine clinical data employed in this study. Our exploratory safety signal analyses may inform future research; however, due to the nature of the EMR data (i.e., diagnoses are mostly not linked to treatments, and primary care doctors may not systematically capture side effects), the results cannot be used to establish safety profiles for a specific drug or medicinal product. There is a risk of misclassification, as routine care data and EMR might vary in the accuracy of their recordings. It is noteworthy, however, that the validity, robustness (as determined by sensitivity analyses), and representativeness of the DA have been demonstrated in previous studies [48,49] and deemed useful for real-world evidence research. One might expect this misclassification bias to be non-differential between cohorts (i.e., cohorts are equally likely to have inaccurately recorded entries and records); thus, the misclassification bias should affect cohorts equally and, therefore, cannot account for the pattern of results observed here. More specifically, it is plausible to assume that coding accuracy variability would be equally distributed across patients. This means an Nx4 patient would have the same probability of an inaccurate EMR record as a BZD or Z-drug patient, especially when matched on age, co-morbidities, and number of clinician visits. With an equally distributed misclassification bias, the effects would be non-differential, typically not resulting in an overestimation of an association, but rather in a bias towards the null. Consequently, the expected misclassification bias in this study might have resulted in an underestimation of the differences between groups and their association with study outcomes. The generalizability of the findings to sleep disorder patients in general is limited in regard to regional applicability, i.e., the situation in Germany; it should be considered in the context of routine care in primary physician practices. It is unknown whether and to what extent patients have received specialist care, which would be especially relevant for more severe sleep disorder patients. This implies the possibility of a sampling bias.

#### 4.4.5. Limitations of Statistical Methods

In the present study, a matched-pairs design was employed, which is considered a robust technique for reducing the impact of confounders. However, this design has its limitations. For example, some patients may drop out of a study, and matched-pairs design can lead to overmatching bias. Nevertheless, in our study, only a small number of variables were used for matching, reducing the likelihood of overmatching bias. Potential confounders, uncontrolled in this study, include diagnosis severity and or socio-economic status (SES), which could affect the probability of an Nx4 prescription. When we included additional potential confounders in the matching procedures during secondary analyses (such as antidepressant prescription prior to the index date or prescription practice preferences), the results for the association between Nx4 and recurrent sleep disorders remained robust. Overall, these additional adjustments did not affect the link between Nx4 and depression diagnosis, except for practice prescription behavior. This suggests that the likelihood of receiving an Nx4 prescription (as measured by practice prescription preferences) might mediate the moderate association observed between Nx4 and a lower risk of depression diagnosis during follow-up. Disease severity might also play a role, as individuals with less severe symptoms might seek out practices more likely to prescribe natural medicinal products. These results further emphasize the importance of capturing disease severity in future studies, either by including results from diagnostic interviews, physician reports, or additional EMR records from specialist physicians or hospital visits, all of which were unavailable in this dataset. Indeed, prior studies have indicated a positive association between higher education levels and the use of homeopathy [68], and SES is recognized as a predictor of sleep disorders and depression in Germany and globally [69,70,71,72,73]. Thus, SES might be a confounder in the present study. However, the IQVIA DA database, like many EMR databases, does not include SES information, so this variable could not be accounted for. If higher socioeconomic status was (directly or indirectly) protective against sleep disorders and depression, and if higher SES is associated with the use of natural medicinal products, our results might overestimate the association between Nx4 and the study outcomes. Additionally, as with all observational studies using secondary data, our results might be affected by residual confounding. Future studies, whether enriched non-interventional observational studies or case-control studies, should include SES as a potential confounder in study design, for example, as a covariate or a matching criterion. Finally, while real-world evidence can provide valuable insights into the usage and potential risks of medicinal products, data quality, confounding, and bias remain fundamental issues that need to be carefully considered when evaluating results from such data sources [74]. This current study cannot establish causality but represents an observational association study, indicating a link between Nx4 and sleep disorder recurrence and depression. Future studies are needed to explore the association between Nx4 prescription and sleep and depression disorders while considering potential confounders such as disorder severity and SES.

## 5. Conclusions

Taken together, our analyses suggest that the natural medicinal product Nx4 might be associated with lower levels of sleep disorder chronification and lower associated depression prevalence compared to Z-drugs and BZD, while there is no overall distinction between Nx4, Z-drugs, and BZD in terms of safety profile. Although the current study is limited in establishing a causal relationship between Nx4 prescription and health outcomes due to inherent study design limitations, it still adds evidence supporting the use of natural medicinal products as alternative treatment options to common prescription hypnotics, whose shortcomings are well known. 

## Figures and Tables

**Table 1:** Patient characteristics by study cohort after 1:1 matching.

Patients with a Prescription of	Nx4	Z-Drug	*p*-Value	Nx4	BZD	*p*-Value
*n*	8594	8594		7779	7779	
Variables included in matching						
Age; Mean (SD)	48.0 (18.5)	48.0 (18.5)	1.000	50.0 (17.9)	50.0 (17.9)	1.000
≤30 years; *n* (%)	1871 (21.8)	1871 (21.8)	1.000	1308 (16.8)	1308 (16.8)	1.000
31–40 years; *n* (%)	1338 (15.6)	1338 (15.6)	1.000	1207 (15.5)	1207 (15.5)	1.000
41–50 years; *n* (%)	1611 (18.8)	1611 (18.8)	1.000	1545 (19.9)	1545 (19.9)	1.000
51–60 years; *n* (%)	1651 (19.2)	1651 (19.2)	1.000	1604 (20.6)	1604 (20.6)	1.000
61–70 years; *n* (%)	913 (10.6)	913 (10.6)	1.000	913 (11.7)	913 (11.7)	1.000
>70 years; *n* (%)	1210 (14.1)	1210 (14.1)	1.000	1202 (15.5)	1202 (15.5)	1.000
Sex: female; *n* (%)	5372 (62.5)	5372 (62.5)	1.000	4904 (63.0)	4904 (63.0)	1.000
Depression; *n* (%)	1281 (14.9)	1281 (14.9)	1.000	1236 (15.9)	1236 (15.9)	1.000
Anxiety disorder; *n* (%)	436 (5.1)	436 (5.1)	1.000	512 (6.6)	512 (6.6)	1.000
Severe stress and adjustment disorders; *n* (%)	1360 (15.8)	1360 (15.8)	1.000	1098 (14.1)	1098 (14.1)	1.000
Number of consultations during the 12 months of follow-up; mean (SD)	4.9 (5.0)	4.9 (5.0)	0.642	4.9 (5.0)	4.9 (5.0)	0.563
Further variables						
Back pain; *n* (%)	1956 (22.8)	1766 (20.6)	<0.001	1812 (23.3)	1637 (21.0)	<0.001
Heart disease; *n* (%)	942 (11.0)	906 (10.5)	0.375	906 (11.7)	934 (12.0)	0.487
Diabetes; *n* (%)	460 (5.4)	589 (6.9)	<0.001	453 (5.8)	565 (7.3)	<0.001
Chronic bronchitis/COPD; *n* (%)	326 (3.8)	326 (3.8)	1.000	310 (4.0)	391 (5.0)	0.002
Cancer; *n* (%)	232 (2.7)	409 (4.8)	<0.001	228 (2.9)	417 (5.4)	<0.001

**Table 2:** Association between Nx4 prescription and probability of recurrent sleep disorder diagnosis within 30–365 days after the index date versus Z-drugs and BZD in multivariable-adjusted Cox regression models.

	Nx4 versus Z-Drugs		Nx4 versus BZD	
Subgroup	Hazard Ratio (95%CI)	*p*-Value	Hazard Ratio (95%CI)	*p*-Value
Total	0.65 (0.60–0.70)	<0.001	0.85 (0.79–0.93)	<0.001
≤30 years	0.70 (0.58–0.85)	<0.001	0.85 (0.66–1.09)	0.194
31–40 years	0.63 (0.50–0.79)	<0.001	0.63 (0.50–0.80)	<0.001
41–50 years	0.71 (0.59–0.85)	<0.001	0.99 (0.81–1.20)	0.910
51–60 years	0.57 (0.48–0.68)	<0.001	0.78 (0.65–0.94)	0.010
61–70 years	0.64 (0.54–0.74)	<0.001	0.98 (0.73–1.11)	0.316
>70 years	0.64 (0.52–0.77)	<0.001	0.88 (0.75–1.04)	0.135
Men	0.74 (0.66–0.84)	<0.001	0.84 (0.74–0.96)	0.010
Women	0.60 (0.55–0.66)	<0.001	0.86 (0.76–0.95)	0.004

**Table 3:** Association between Nx4 prescription and probability of a depression diagnosis within 30–365 days after the index date versus Z-Drugs and BZD in multivariable-adjusted Cox regression models.

	Nx4 versus Z-Drugs		Nx4 versus BZD	
Subgroup	Hazard Ratio (95%CI)	*p*-Value	Hazard Ratio (95%CI)	*p*-Value
Total	0.90 (0.83–0.98)	0.020	0.89 (0.82–0.97)	0.009
≤30 years	0.78 (0.64–0.96)	0.019	0.98 (0.77–1.23)	0.831
31–40 years	0.92 (0.74–1.15)	0.460	0.85 (0.68–1.08)	0.181
41–50 years	0.86 (0.71–1.05)	0.148	0.75 (0.62–0.92)	0.005
51–60 years	0.92 (0.77–1.11)	0.384	0.82 (0.69–0.98)	0.032
61–70 years	0.97 (0.75–1.26)	0.833	0.90 (0.69–1.16)	0.395
>70 years	1.01 (0.82–1.24)	0.931	1.16 (0.93–1.44)	0.195
Men	0.88 (0.76–1.02)	0.095	0.91 (0.78–1.07)	0.258
Women	0.91 (0.82–1.01)	0.084	0.88 (0.79–0.98)	0.016

## Data Availability

The data and the code used for this study are available from C.V. upon reasonable request.

## Supplementary Materials

The following supporting information can be downloaded at: https://www.mdpi.com/article/10.3390/healthcare12141413/s1. Supplementary Table S1: Association between Nx4 prescription and the prevalence of fractures within 1–365 days after the index date versus Z-drugs and benzodiazepines (BZD) in multivariable-adjusted Cox regression models. Supplementary Table S2: Association between Nx4 prescription and the prevalence of lower respiratory tract infections such as bronchitis and pneumonia within 1–365 days after the index date versus Z-drugs and benzodiazepines (BZD) in multivariable-adjusted Cox regression models. Supplementary Table S3: Association between Nx4 prescription and the prevalence of symptoms involving the digestive system and the abdomen within 1–365 days after the index date versus Z-drugs and benzodiazepines (BZD) in multivariable-adjusted Cox regression models. Supplementary Table S4: Association between Nx4 prescription and the prevalence of symptoms involving cognition and perception within 1–365 days after the index date versus Z-drugs and benzodiazepines (BZD) in multivariable-adjusted Cox regression models. Supplementary Table S5: Association between Nx4 prescription and probability of a recurrent sleep disorder diagnosis within 30–365 days after the index date versus Z-drugs and benzodiazepines (BZD) in multivariable-adjusted Cox regression models, with subsequent inclusion of additional matching variables in using a nearest-neighbor propensity score matching algorithm. Supplementary Table S6: Association between Nx4 prescription and probability of a depression diagnosis within 30–365 days after the index date versus Z-drugs and benzodiazepines (BZD) in multivariable-adjusted Cox regression models, with subsequent inclusion of additional matching variables in using a nearest-neighbor propensity score matching algorithm.

## Abbreviations

The following abbreviations are used in this manuscript:

BZD	Benzodiazepines
CBT	Cognitive behavioral therapy
CI	Confidence interval
COPD	Chronic obstructive pulmonary disease
DA	Disease analyzer
EMR	Electronic medical record
GP	General practitioner
HR	Hazard ratio
ICD	International Statistical Classification of Diseases and Related Health Problems
*n*	Number of observations
Nx4	Neurexan
OTC	Over-the-counter
SD	Standard deviation
SES	Socio-economic status
SMD	Standardized mean difference
