# Supplementary material for: Neurexan Prescription Is Associated with Lower Risk of Sleep Disorder Recurrence and Depression Prevalence as Compared to Z-Drugs and Benzodiazepines: A Retrospective Database Analysis in Germany
Source: Healthcare (Basel). 2024 Jul 16;12(14):1413. doi: 10.3390/healthcare12141413 (PMC11276089; doi:10.3390/healthcare12141413)
Supplement: Supplementary file 1 [file healthcare-12-01413-s001.zip › healthcare-3055799-supplementary.pdf]

Supplementary Table S1: Association between Nx4 prescription and the prevalence of fractures within 1–365 days after the index date versus Z-drugs and benzodiazepines (BZD) in multivariable-adjusted Cox regression models.

	Proportion of affected patients on the index date		Nx4 *vs*. Z-drugs (Ref.)		Proportion of affected patients on the index date		Nx4 *vs*. BZD (Ref.)	
Subgroup	% Nx4	% Z-drugs	Hazard Ratio (95% CI)	P-value	% Nx4	% BZD	Hazard Ratio (95% CI)	P-value
Total	2.4	2.5	0.93 (0.76–1.12)	0.436	2.3	2.5	0.93 (0.76–1.15)	0.514
≤30 years	1.3	0.9	1.57 (0.84–2.94)	0.161	1.0	1.3	0.78 (0.38–1.60)	0.496
31–40 years	0.9	1.1	0.82 (0.38–1.78)	0.623	0.9	1.5	0.61 (0.29–1.28)	0.190
41–50 years	1.6	1.7	0.93 (0.54–1.60)	0.793	1.2	1.2	1.03 (0.54–1.97)	0.924
51–60 years	2.7	2.6	0.99 (0.65–1.51)	0.969	2.7	2.1	1.28 (0.81–2.02)	0.294
61–70 years	3.1	3.1	1.00 (0.59–1.69)	0.992	3.2	2.4	1.31 (0.74–2.29)	0.354
>70 years	5.5	7.4	0.75 (0.54–1.03)	0.074	5.5	7.2	0.74 (0.54–1.02)	0.068

Supplementary Table S2: Association between Nx4 prescription and the prevalence of lower respiratory tract infections such as bronchitis and pneumonia within 1–365 days after the index date versus Z-drugs and benzodiazepines (BZD) in multivariable-adjusted Cox regression models.

	Proportion of affected patients on the index date		Nx4 *vs*. Z-drugs (Ref.)		Proportion of affected patients on the index date		Nx4 *vs*. BZD (Ref.)	
Subgroup	% Nx4	% Z-drugs	Hazard Ratio (95% CI)	P-value	% Nx4	% BZD	Hazard Ratio (95% CI)	P-value
Total	11.0	11.0	0.93 (0.76–1.12)	0.436	10.7	11.5	0.93 (0.85–1.02)	0.134
≤30 years	11.0	11.9	0.92 (0.76–1.11)	0.364	8.9	9.7	0.93 (0.72–1.20)	0.585
31–40 years	10.5	10.9	0.95 (0.75–1.19)	0.640	10.7	10.1	1.05 (0.82–1.35)	0.702
41–50 years	11.7	11.7	0.99 (0.81–1.21)	0.925	11.7	12.2	0.94 (0.77–1.16)	0.580
51–60 years	11.2	11.4	1.00 (0.82–1.22)	0.970	11.4	13.9	0.79 (0.65–0.97)	0.022
61–70 years	9.2	9.1	1.01 (0.75–1.36)	0.936	9.1	12.8	0.69 (0.52–0.92)	0.011
>70 years	12.1	9.8	1.26 (0.99–1.61)	0.064	12.0	9.9	1.27 (1.00–1.63)	0.053

Supplementary Table S3: Association between Nx4 prescription and the prevalence of symptoms involving the digestive system and the abdomen within 1–365 days after the index date versus Z-drugs and benzodiazepines (BZD) in multivariable-adjusted Cox regression models.

	Proportion of affected patients on the index date		Nx4 *vs*. Z-drugs (Ref.)		Proportion of affected patients on the index date		Nx4 *vs*. BZD (Ref.)	
Subgroup	% Nx4	% Z-drugs	Hazard Ratio (95% CI)	P-value	% Nx4	% BZD	Hazard Ratio (95% CI)	P-value
Total	12.5	11.7	1.07 (0.99–1.17)	0.107	12.2	11.8	1.05 (0.95–1.15)	0.339
≤30 years	14.4	13.0	1.12 (0.94–1.33)	0.215	13.2	13.0	1.01 (0.81–1.25)	0.947
31–40 years	10.6	9.6	1.10 (0.87–1.40)	0.420	10.9	9.5	1.16 (0.90–1.49)	0.246
41–50 years	9.6	9.6	1.05 (0.83–1.31)	0.704	9.6	9.6	1.02 (0.81–1.28)	0.892
51–60 years	11.8	9.7	1.23 (0.99–1.52)	0.057	11.5	9.9	1.18 (0.96–1.47)	0.124
61–70 years	11.5	11.1	1.07 (0.81–1.41)	0.618	11.5	12.6	0.97 (0.74–1.26)	0.799
>70 years	17.4	18.4	0.94 (0.78–1.14)	0.532	17.0	17.6	0.96 (0.79–1.16)	0.644

Supplementary Table S4: Association between Nx4 prescription and the prevalence of symptoms involving cognition and perception within 1–365 days after the index date versus Z-drugs and benzodiazepines (BZD) in multivariable-adjusted Cox regression models.

	Proportion of affected patients on the index date		Nx4 *vs*. Z-drugs (Ref.)		Proportion of affected patients on the index date		Nx4 *vs*. BZD (Ref.)	
Subgroup	% Nx4	% Z-drugs	Hazard Ratio (95% CI)	P-value	% Nx4	% BZD	Hazard Ratio (95% CI)	P-value
Total	5.4	3.9	1.41 (1.22–1.62)	<0.001	5.4	4.4	1.05 (0.95–1.15)	0.339
≤30 years	4.1	2.8	1.47 (1.03–2.10)	0.032	3.4	3.4	1.02 (0.81–1.25)	0.945
31–40 years	3.4	2.4	1.38 (0.88–2.18)	0.161	3.4	3.7	1.16 (0.90–1.49)	0.246
41–50 years	3.6	2.4	1.50 (1.00–2.25)	0.053	3.6	2.8	1.02 (0.81–1.28)	0.892
51–60 years	5.3	3.3	1.63 (1.16–2.29)	0.005	5.2	3.7	1.18 (0.96–1.47)	0.124
61–70 years	5.3	3.5	1.50 (0.95–2.36)	0.079	5.5	4.3	0.97 (0.74–1.26)	0.799
>70 years	12.5	10.3	1.21 (0.96–1.54)	0.113	12.2	9.4	0.96 (0.79–1.16)	0.644

Supplementary Table S5: Association between Nx4 prescription and probability of a recurrent sleep disorder diagnosis within 30–365 days after the index date versus Z-drugs and benzodiazepines (BZD) in multivariable-adjusted Cox regression models, with subsequent inclusion of additional matching variables in using a nearest-neighbor propensity score matching algorithm.

	Nx4 *vs*. Z-drugs (Ref.)		Nx4 *vs*. BZD (Ref.)	
Sequentially included variables in the matching algorithm	Hazard Ratio (95% CI)	P-value	Hazard Ratio (95% CI)	P-value
Age, sex, depression, anxiety disorder, reaction to severe stress/adjustment disorder, number of medical consultations	0.65 (0.60–0.70)	<0.001	0.85 (0.79–0.93)	<0.001
Age, sex, depression, anxiety disorder, reaction to severe stress/adjustment disorder, number of medical consultations, diabetes	0.65 (0.60-0.70)	<0.001	0.81 (0.75-0.88)	<0.001
Age, sex, depression, anxiety disorder, reaction to severe stress/adjustment disorder, number of medical consultations, diabetes, COPD	0.64 (0.60-0.69)	<0.001	0.82 (0.76-0.89)	<0.001
Age, sex, depression, anxiety disorder, reaction to severe stress/adjustment disorder, number of medical consultations, diabetes, COPD, back pain	0.67 (0.62-0.72)	<0.001	0.81 (0.75-0.87)	<0.001
Age, sex, depression, anxiety disorder, reaction to severe stress/adjustment disorder, number of medical consultations, diabetes, COPD, back pain, cancer	0.65 (0.60-0.70)	<0.001	0.81 (0.75-0.87)	<0.001
Age, sex, depression, anxiety disorder, reaction to severe stress/adjustment disorder, number of medical consultations, diabetes, COPD, back pain, cancer, heart diseases	0.66 (0.62-0.71)	<0.001	0.83 (0.77-0.89)	<0.001
Age, sex, depression, anxiety disorder, reaction to severe stress/adjustment disorder, number of medical consultations, diabetes, COPD, back pain, cancer, heart diseases, antidepressant therapy within 12 months prior to index date	0.68 (0.63-0.73)	<0.001	0.83 (0.77-0.89)	<0.001
Age, sex, depression, anxiety disorder, reaction to severe stress/adjustment disorder, number of medical consultations, diabetes, COPD, back pain, cancer, heart diseases, antidepressant therapy within 12 months prior to index date, and practice prescription behavior*	0.64 (0.59-0.69)	<0.001	0.83 (0.76-0.90)	<0.001

* Practice prescription behavior was estimated based in the number of patients with sleep disorder who received Nx4 versus Z-drugs or benzodiazepine. Proportion of patients per practice was calculated as number of patients who received Nx4 (0%, 1-24%, 25-49%, 50-79% und 80+%).

Supplementary Table S6: Association between Nx4 prescription and probability of a depression diagnosis within 30–365 days after the index date versus Z-drugs and benzodiazepines (BZD) in multivariable-adjusted Cox regression models, with subsequent inclusion of additional matching variables in using a nearest-neighbor propensity score matching algorithm.

	Nx4 *vs*. Z-drugs (Ref.)		Nx4 *vs*. BZD (Ref.)	
Sequentially included variables in the matching algorithm	Hazard Ratio (95% CI)	P-value	Hazard Ratio (95% CI)	P-value
Age, sex, depression, anxiety disorder, reaction to severe stress/adjustment disorder, number of medical consultations	0.90 (0.83–0.98)	0.020	0.89 (0.82–0.97)	0.009
Age, sex, depression, anxiety disorder, reaction to severe stress/adjustment disorder, number of medical consultations, diabetes	0.92 (0.86-0.98)	0.012	0.84 (0.78-0.90)	<0.001
Age, sex, depression, anxiety disorder, reaction to severe stress/adjustment disorder, number of medical consultations, diabetes, COPD	0.93 (0.87-0.99)	0.025	0.85 (0.78-0.91)	<0.001
Age, sex, depression, anxiety disorder, reaction to severe stress/adjustment disorder, number of medical consultations, diabetes, COPD, back pain	0.92 (0.86-0.98)	0.010	0.82 (0.76-0.89)	<0.001
Age, sex, depression, anxiety disorder, reaction to severe stress/adjustment disorder, number of medical consultations, diabetes, COPD, back pain, cancer	0.93 (0.87-0.99)	0.034	0.86 (0.79-0.93)	<0.001
Age, sex, depression, anxiety disorder, reaction to severe stress/adjustment disorder, number of medical consultations, diabetes, COPD, back pain, cancer, heart diseases	0.95 (0.89-1.01)	0.122	0.85 (0.79-0.92)	<0.001
Age, sex, depression, anxiety disorder, reaction to severe stress/adjustment disorder, number of medical consultations, diabetes, COPD, back pain, cancer, heart diseases, antidepressant therapy within 12 months prior to index date	0.94 (0.88-0.99)	0.047	0.87 (0.81-0.94)	0.001
Age, sex, depression, anxiety disorder, reaction to severe stress/adjustment disorder, number of medical consultations, diabetes, COPD, back pain, cancer, heart diseases, antidepressant therapy within 12 months prior to index date, and practice prescription behavior*	0.98 (0.92-1.06)	0.664	1.00 (0.92-1.11)	0.844
